# The prevalence of type 2 diabetes mellitus risk factors among patients at three selected clinics in Windhoek, Namibia

**DOI:** 10.4102/hsag.v31i0.3426

**Published:** 2026-10-09

**Authors:** Lynnete C. Mukonka, Ntombifikile G. Mtshali

**Affiliations:** 1 School of Nursing and Public Health, Faculty of Health Sciences, University of KwaZulu-Natal, Durban, South Africa

**Keywords:** type 2 diabetes mellitus, risk factors, patients, prevention, prevalence

## Abstract

**Background:**

Worldwide, there is an increase in the prevalence of type 2 diabetes mellitus (T2DM). Identification of the most prevalent risk factors within different communities assists in taking measures to curb the projected rise in the prevalence of T2DM in the years to come.

**Aim:**

The aim of the study was to determine the prevalence of T2DM risk factors among patients at selected clinics in Windhoek, Namibia, in order to design data-driven preventive strategies.

**Setting:**

The study was conducted at three clinics in Windhoek, Namibia.

**Methods:**

This article uses data taken from a mixed-methods study aimed at developing a lifestyle modification programme for the prevention of T2DM, involving 673 participants. Out of these 673 participants, data from 634 non-diabetic respondents were used. A questionnaire was used to collect data, and the American Diabetes Association (ADA) Diabetes risk test was also used to assess the risk levels among the non-diabetic participants. Descriptive statistics were used to analyse the data.

**Results:**

The most prevalent factor was having a family history of hypertension (258; 42.3%), then history of T2DM (267; 39.7%) and age (223; 33.1%). Other factors identified were random blood glucose levels and waist circumference. Most of the respondents (481; 75.9%) had an overall low risk of developing T2DM, while 98 (15.5%) respondents had a high risk of developing T2DM.

**Conclusion:**

Although the most prevalent factors were non-modifiable factors, the results of the ADA risk test indicate a combination of both modifiable and non-modifiable risk factors. There is a need for preventive strategies, and further research on cost-effective measures is also recommended.

**Contribution:**

The study shows the need for T2DM preventive measures and further research.

## Introduction

The prevalence of diabetes mellitus (DM) worldwide has been increasing and is projected to increase by 45% by the year 2050 (International Diabetes Federation [IDF] 2025). In the earlier years, DM was seen as a disease of the populations in developed countries; however, as the years progressed, this trend is changing as developing countries are also having a high prevalence of DM (Hossain, Al-Mamun & Islam 2024).

According to the IDF 11th Edition (2025), the prevalence of DM worldwide as of 2024 was 588.7 million, and it is projected that the prevalence of DM will increase to 852.5 million by 2050. In Africa, prevalence was 24.6 million, and the projection for 2050 would be a 142% increase to 59.5 million. Type 2 diabetes mellitus (T2DM) accounts for 90% of the prevalence (IDF 2025).

Sub-Saharan African (SSA) countries like Namibia are expected to have a 142% rise in the prevalence of T2DM by 2050 (IDF 2025). Urbanisation and lifestyle changes when people move from their rural communities, as well as increased exposure to fast foods, are some of the reasons why the prevalence is increasing (Goyal et al. 2020). The current proportion of people in SSA with undiagnosed diabetes is 72.6%, making it the highest worldwide. The lack of infrastructure within healthcare systems and lack of awareness of early symptoms are most likely to be the contributing factors to this (Traoré et al. 2024).

Risk factors for the development of T2DM can be modifiable and non-modifiable. The modifiable factors include being overweight, a sedentary lifestyle, an unhealthy diet, high blood pressure, as well as smoking and consumption of alcohol (Hossain et al. 2024). The modifiable risk factors become the key aspects for the prevention of T2DM. The non-modifiable factors include age, sex and family history of T2DM and hypertension (Hossain et al. 2024).

There are trials that have been conducted to come up with ways of preventing T2DM. There was the 30-year-long Da Qing impaired glucose tolerance (IGT) and Diabetes study, which began in 1986; it was shown that lifestyle intervention reduced the incidence of diabetes (Gong et al. 2020). The same was observed in the Finnish Diabetes Prevention Study (Finnish DPS) from 1993 to 2001. The United States Diabetes Prevention Program (DPP) and the United States Diabetes Prevention Program Outcome Study (DPPOS) showed that lifestyle interventions were more cost-effective than the placebo. Other countries like Australia and Korea have also adapted these diabetes prevention programmes to tailor them to suit their context. In SSA, evidence shows a few programmes or policies regarding the prevention of T2DM (Mukonka, Chironda & Mtshali 2026). In 2024, the World Health Organization (WHO) launched the global monitoring framework on diabetes to assist countries in coming up with interventions and policies for diabetes prevention and care. Other initiatives aimed at the prevention of T2DM launched by the WHO include the WHO’s Global Diabetes Compact, which was launched in 2021 and the five global diabetes coverage targets to be achieved by 2030 (WHO 2024).

The prevalence of risk factors may be a projection of the future burden of T2DM. Therefore, early identification of these factors enables implementation of preventive measures. This study will thus contribute to strategies to curb the projected rise in cases of T2DM.

### Research aim

The aim of the research was to determine the prevalence of T2DM risk factors among patients at three selected clinics in Windhoek in order to design data-driven preventive strategies.

## Research methods and design

### Research design

Informed by a positivist paradigm, the research employed a quantitative approach and provides objective, generalisable insights, which enables the researcher to find patterns and make predictions across large populations (Smith 2024). A cross-sectional survey was used as it enables large population-based data gathering from a group representative of the population of interest, to quantify trends enabling data-driven decisions (Ponto 2015). The results presented are part of the main study aimed at developing a lifestyle modification programme for the prevention of T2DM.

### Setting

The study was conducted at three conveniently selected primary health care (PHC) clinics in Windhoek, Namibia. The clinics were conveniently selected because they are among the clinics with a high catchment area and diverse population. The clinics serve various ethnic groups found in Namibia; the residential areas, to a certain extent, are separated by ethnicity or race.

### Population and sampling strategy

Adults aged 18 years and above who lived within the catchment areas of the clinics were the target population. There were about 30 000 people above the age of 18 years in the catchment areas for the study sites. This report is part of a study aimed at developing a lifestyle modification program for the prevention of T2DM. The sample size for the study was determined by logistic regression and was calculated to be 670, and data were collected from 673 respondents. The respondents included diabetics and non-diabetics; however, for this report on the prevalence of risk factors for T2DM, data from the non-diabetics were considered. Simple random sampling was used to select respondents for the study. A sampling frame was compiled from the daily attendance registers. A list of clients aged 18 years and above who fit the eligibility criteria was used for simple random selection of participants. Data from the diabetics were excluded, as their behaviour at the time of data collection may not reflect the risk behaviour required for this article. Clients aged 18 years and above, attending the outpatients’ clinic at the three clinics were used because they were the accessible population. The ability of the clients to converse in English or Afrikaans or Oshiwambo were also considered for clear communication with the participants.

### Data collection

The data collection instrument was a structured questionnaire which was pre-tested on five clients from the study population who met the eligibility criteria. The five did not form part of the study respondents. There were no adjustments that were made to the questionnaire. The questionnaires were administered by the researcher and the assistants at the three study sites. Data were collected concurrently over 1 month at the three sites, for 6 days per week. The managers at the study sites offered rooms for use by the researcher and the assistants during data collection to ensure privacy, as data were collected from one respondent at a time. The American Diabetes Association (ADA) Diabetes risk test was also used to assess the risk levels among the respondents using the data from the respondents.

### Data analysis

Following data collection, the questionnaires were checked for completeness. The data were coded and entered into Microsoft Excel 2016, then exported to R software for data analysis. Descriptive statistics such as frequencies, percentages, means and interquartile ranges were used to summarise demographic data and the study variables on the risk factors for T2DM.

### Ethical considerations

Ethical clearance to conduct this study was obtained from the University of KwaZulu-Natal Biomedical Research Ethics Committee (No. BREC/00003860/2022). The Ministry of Health and Social Services for Namibia gave the gatekeeper’s permission for the research to be conducted with the approval number 17/3/3/LCM001. Written consent was obtained from the respondents after they were given information about the study, which included the main aim of the study and what was expected from them. Data collection was done in private rooms, where respondents were invited individually to ensure anonymity and confidentiality. To ensure anonymity, no identifying information was included in the completed questionnaires, and the signed consent forms were kept in separate folders from the completed questionnaires. For confidentiality, data were not shared with anyone outside the research team, except in the cases where the blood glucose level was elevated and the respondents needed to be referred for management of the elevated blood glucose levels.

## Results

### Demographic information

This report focuses on 634 respondents, who were non-diabetics and were attending the outpatient clinics at the three study sites. The respondents were mostly Christians (*n* = 619; 97.6%), and most of them had attained secondary education or higher (*n* = 571; 90.1%). The majority were also single (*n* = 453; 71.5%), followed by those who were married or living together with their partners (*n* = 157; 24.8%), and those who were separated or divorced or other were 10 (1.5%). Nearly all of the respondents were Africans (*n* = 595; 93.8%), and other races were white, Indian, coloured and other, who were 4 (0.6%), 1 (0.2%), 32 (5%) and 2 (0.3%), respectively. The unemployed were 261 (41.2%), the formally employed were 245 (38.6%), 36 (5.7%) were casually employed, and 82 (12.9%) and 10 (1.6%) were self-employed and retired, respectively. In terms of gender, there were 321 (50.6%) females and 313 (49.4%) males. The respondents’ ages ranged between 18 years and 78 years, with a mean age of 43.9 years (s.d. 13.5). Table 1 shows the demographic characteristics of the respondents.

**TABLE 1:** Demographic information of respondents (*N* = 634).

Demographic information	Frequency (*n*)	%	Median	Q1–Q3	Min–Max
**Religion**					
Christianity	619	97.6	-	-	-
Other	10	1.6	-	-	-
Islam	3	0.5	-	-	-
Traditional	2	0.3	-	-	-
**Education level**					
No schooling	18	2.8	-	-	-
Primary	45	7.1	-	-	-
Secondary	391	61.7	-	-	-
Tertiary	180	28.4	-	-	-
**Marital status**					
Single	453	71.5	-	-	-
Married or living together	157	24.8	-	-	-
Widowed	14	2.2	-	-	-
Separated or divorced	6	0.9	-	-	-
Other	4	0.6	-	-	-
**Race**					
African people	595	93.8	-	-	-
White people	4	0.6	-	-	-
Indian people	1	0.2	-	-	-
Coloured people	32	5.0	-	-	-
Other	2	0.3	-	-	-
**Employment status**					
Unemployed	261	41.2	-	-	-
Formally employed	245	38.6	-	-	-
Casually employed	36	5.7	-	-	-
Self-employed	82	12.9	-	-	-
Retired	10	1.6	-	-	-
**Gender**			-	-	-
Female	321	50.6	-	-	-
Male	313	49.4	-	-	-
**Age (years)**					
Mean ± s.d. (CV%)	-	-	-	-	-
Median (Q1–Q3)	-	-	34.0	26.0–42.8	-
*n* (Min–Max)	634	-	-	-	18.0–78.0

CV, coefficient of variation.

### Prevalence of type 2 diabetes mellitus risk factors

Table 2 shows the prevalence of risk factors for T2DM among the participants. Most of the female respondents (*n* = 296; 92.2%) had no history of gestational diabetes, and 25 (7.8%) had had gestational diabetes. Slightly more than half of the respondents, 394 (62.1%), did not have a family history of DM, while 240 (37.9%) had a family history of DM. There were 258 (40.7%) respondents with a history of hypertension, while 376 (59.3%) did not have a history of hypertension. Close to half of the respondents were obese and overweight (*n* = 306; 48.2%), and the rest had either normal body mass index (BMI) (*n* = 283; 44.6%) or were underweight (*n* = 45; 7.1%).

**TABLE 2:** Prevalence of type 2 diabetes mellitus risk factors (*N* = 634).

Risk factor	Frequency (*n*)	%
**Gestational diabetes mellitus**		
No	296	92.2
Yes	25	7.8
**Diabetes mellitus history**		
No	394	62.1
Yes	240	37.9
**Hypertensive**		
No	376	59.3
Yes	258	40.7
**BMI**		
Underweight	45	7.1
Normal	283	44.6
Overweight	167	26.3
Obese	139	21.9
**Random blood glucose levels**		
Excluded	466	73.5
Inconclusive	164	25.9
Diabetic	4	0.6
**Waist circumference levels**		
Normal	433	68.3
Increased risk	201	31.7
**Age**		
Normal	437	68.9
Increased risk	197	31.1

BMI, body mass index.

Four (0.6%) respondents had high random blood sugar (RBS) levels. Those who had inconclusive RBS were 164 (25.9%), and 466 (73.5%) had normal RBS. As for waist circumference, 433 (68.3%) respondents had normal waist circumference, and 201 (31.7%) respondents had waist circumference indicating increased risk for development of T2DM. Four hundred and thirty-seven (*n* = 437; 68.9%) respondents were within the normal age range, and 197 (31.1%) respondents were in the increased-risk age group.

### Diabetes mellitus risk levels

The DM risk levels for the 634 non-diabetic respondents were calculated using the ADA diabetes risk test, and the results were as shown in Figure 1. Fifty-five (*n* = 55; 8.7%) respondents were at no risk, 481 (75.9%) were at low risk, and 98 (15.5%) were at high risk of developing DM.

**FIGURE 1:**
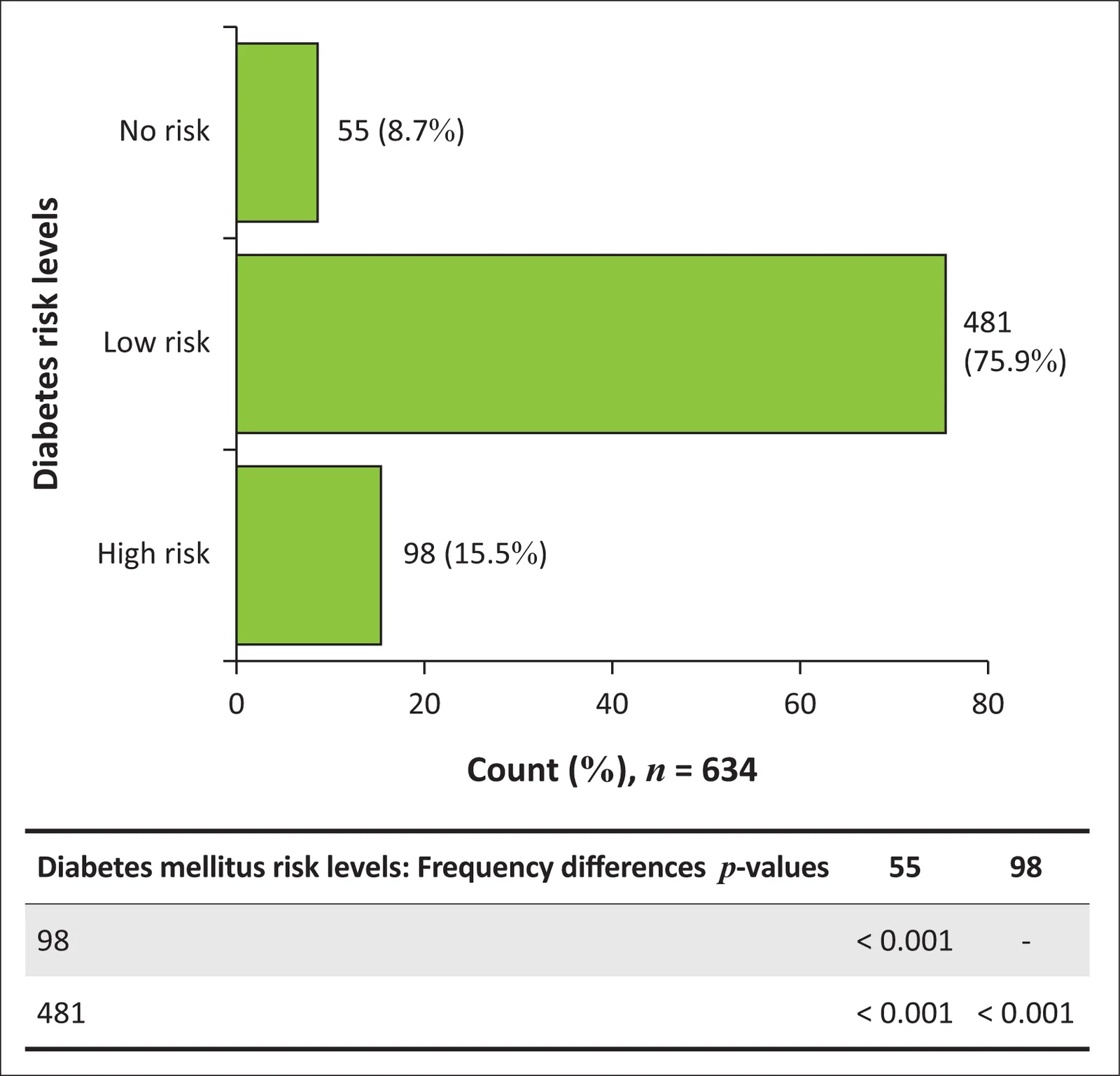
Diabetes mellitus risk levels among the respondents (*N* = 634).

### Prevalence of other risk factors for type 2 diabetes mellitus

There are other behaviour-related risk factors for T2DM, of which their prevalence among the study respondents is shown in Table 3. The most prevalent factor was alcohol consumption (*n* = 267; 42.1%). This was followed by lack of exercise (*n* = 214; 33.8%), smoking (*n* = 70; 11.0%) and non-consumption of fruits and vegetables (*n* = 35; 5.5%).

**TABLE 3:** Prevalence of other risk factors.

Other risk factors	Frequency (*n*)	%
**Exercise**		
No	214	33.8
Yes	420	66.2
**Fruit and vegetable consumption**		
No	35	5.5
Yes	599	94.5
**Smoker**		
No	564	89.0
Yes	70	11.0
**Alcohol consumption**		
No	367	57.9
Yes	267	42.1

## Discussion

This study aimed to determine the prevalence of risk factors for T2DM among outpatients at three selected clinics in Windhoek. The total number of respondents included in this report was 634, who were non-diabetic patients.

It is estimated that 55% – 64% of the adult population may have undiagnosed diabetes (IDF 2025). Although the study did not conduct diagnostic tests for diabetes, there were some respondents who were non-diabetic but had high RBS levels. These could have had undiagnosed T2DM or pre-diabetes. This indicates the need for screening of patients to ensure early detection and management of T2DM. As indicated earlier, there are studies that have been done using lifestyle modification and/or Metformin, which have proven that progression to T2DM can be delayed or regression may occur if treatment is initiated early (The Diabetes Prevention Program Research Group 2012). The WHO also encourages countries to take measures or have initiatives, including policies that are contextually relevant to their countries (WHO 2024). were referred for further management. This may be an indication for further research to assess the levels of prediabetes and diabetes in Windhoek, Namibia.

Among the demographic characteristics, age was a significant factor (*p* < 0.001) for the development of T2DM. The age of onset for T2DM is usually after 45 years; however, this has been decreasing as younger people are getting diagnosed with T2DM (ADA 2025). According to the ADA, in 2021, more than 90 million people aged 18 years and above had prediabetes. A study that examined the global burden of early-onset T2DM from the years 1990 to 2021 indicated that the incidence and prevalence of early-onset T2DM were rising significantly (Zhou et al. 2025). This could be contributed by increasing prevalence of risk factors among young persons (Bo et al. 2018). Some prospective studies showed that individuals with early-onset T2DM even had a higher risk of complications (Lin et al. 2024; Middleton et al. 2020). At an older age, there is either an insufficient quantity of insulin or an inability of insulin to regulate blood glucose levels, leading to T2DM. In the study, the mean age of the respondents was 44 years (s.d. 13.5), which implies that most of the respondents were in the age range that is at risk of developing late-onset T2DM. Several studies conducted in Africa showed that age is associated with an increased risk of T2DM (Chikwati et al. 2025; Issaka et al. 2022, 2023). This underscores the need for strategies for prevention of T2DM.

In this study, family history of DM had a prevalence of 37.9% among the non-diabetics and 69.2 % among the diabetics and was shown to be a significant factor (*p* < 0.001). Type 2 diabetes mellitus is considered to be a result of both genetic and environmental exposure (Li et al. 2020). Therefore, having first-degree relatives who had T2DM could increase the risk of developing the condition. However, family history is a non-modifiable risk factor for T2DM, but measures can still be taken to reduce the occurrence of modifiable factors.

A study conducted in China showed that hypertension in persons above 45 years influenced the risk of T2DM (Ma et al. 2025), and this concurs with the results from this study, where hypertension was shown to be a significant factor (*p* < 0.001). Another study done in Korea showed that hypertension and even prehypertension are significantly associated with the development of diabetes (Kim et al. 2015). This indicates, therefore, the need to prevent or control hypertension using both pharmacological and non-pharmacological interventions to prevent or delay the development of T2DM. In some cases, cardiovascular diseases may occur as complications of T2DM (Middleton et al. 2020), and hypertension may manifest as one of the signs of these complications. Insulin resistance is also linked to the development of hypertension and other cardiovascular diseases (Kim et al. 2015).

Random blood glucose levels were checked among the respondents during data collection, and the chi-square test showed that it was a significant risk factor for the development of T2DM (*p* < 0.001). Although RBS is not a diagnostic test for T2DM, it may indicate the presence of prediabetes or T2DM (Mourougavelou & Chowdhury 2023); therefore, a need for further testing to confirm the diagnosis is recommended. As RBS shows the blood glucose levels at that particular point in time, it can be used as a starting point for early detection of prediabetes or T2DM. This test can thus be used at PHC facilities such that those with abnormal findings may then be referred for further investigations and management.

In Burkina Faso, a study was conducted to estimate the prevalence of undiagnosed DM and its associated factors. The researchers found that there were increased chances of having DM when the age is more than 35 years, family history of DM, being obese or overweight and having hypertension (Traoré et al. 2024). Although the current study did not look at the association of the risk factors with DM, the implications could be the same as the factors stated in the study by Traoré et al (2024) were also seen in the respondents. A retrospective cohort study conducted in Japan also revealed an association between central obesity and the development of T2DM. There was a 72% risk of developing T2DM in individuals with central obesity compared to those with general obesity (Cao et al. 2022). The current study also showed that some respondents had higher-than-normal waist circumference. Therefore, there is a need for screening tools that include all factors to facilitate prevention of T2DM.

Other factors such as BMI, history of GDM, lack of exercise, vegetable and fruit consumption, smoking and alcohol intake did not show significant results in the study. However, various sources in the literature indicate that they also contribute to the development of T2DM (Lovic et al. 2020; Ma et al. 2025; Mourougavelou & Chowdhury 2023).

Using the ADA Type 2 Diabetes Risk Test (ADA n.d.), most of the respondents (*n* = 481; 75.9%) had a low risk, and 98 (15.5%) had a high risk of developing T2DM. This shows that if measures are not taken to return to the no-risk category, chances are high that those with a current low risk may progress to high risk and even develop T2DM. Several studies, including a study done in Egypt, tested and found that the ADA Type 2 Diabetes risk test to be valid and reliable (Abdelmoniem et al. 2024). This test can therefore be used as a screening tool for T2DM risk and thus facilitate implementation of preventive measures. The current treatment guidelines or health policies available in Namibia lack strategies for the prevention of T2DM; hence the need for such guidelines and health policies.

## Conclusion

The findings of the study showed that generally the prevalence of T2DM risk factors was low; however, there is still a need for early identification and implementation of preventive measures where possible to curb the rise in the prevalence of T2DM. At the clinics, there is a need for resources, both material and human, to ensure that screening activities are conducted for all patients. The Ministry of Health should conduct policy reviews to ensure measures for the prevention of T2DM are included and should also ensure the availability of resources for mass education campaigns. The incorporation of T2DM screening activities in other PHC activities may improve early detection as well as awareness among communities. As the study reported on clinic-based samples, they could be different from the community population; therefore, further research is recommended to identify the factors across a larger population group as well as to identify cost-effective measures for diagnosis and prevention of T2DM.
